# Prevalence of bovine fascioliasis and economic impact associated with liver condemnation in abattoirs in Mongu district of Zambia

**DOI:** 10.1186/s12917-019-1777-0

**Published:** 2019-01-21

**Authors:** Stanley S. Nyirenda, Matthews Sakala, Lennon Moonde, Edgar Kayesa, Paul Fandamu, Fredrick Banda, Yona Sinkala

**Affiliations:** 1 Central Veterinary Research Institute, P.O. BOX 33980 Lusaka, Balmoral Zambia; 2 Mongu Veterinary Regional Diagnostic Laboratory, Mongu, Zambia; 3 Department of Veterinary Services, Mulungushi House, Lusaka, Zambia

**Keywords:** Bovine, Fascioliasis, Economic loss, *Lymnaea*, Zambia

## Abstract

**Background:**

Fascioliasis is a trematode zoonotic snail-borne disease of public health and economic importance. The disease causes liver damage and is hardly recognized by medical personnel hence, is rarely considered as the differential diagnosis. In animals, the disease leads to mortalities, growth retardation, drop in livestock production and condemnation of the infected livers during meat inspection.

The cross-sectional study was conducted from 2013 to 2017 in abattoirs in Mongu district, Western province of Zambia. Each selected carcass was examined macroscopically for bovine fascioliasis by dissecting the liver and checking for adult liver flukes. Infested and condemned livers were weighed and incinerated.

**Results:**

A total of 69,152 carcasses with their livers was examined at the abattoirs for adult *Fasciola* worms and 44,511 (64.4%) were positive. According to the intensity of pathological lesions, 55.3% constituted severely affected livers, 30.3% were moderately affected livers and 14.4% were lightly affected livers. Our observation revealed that the most prevalent liver fluke identified was *Fasciola gigantica* (56.1%) and it mostly affected the poor body conditioned animals (71.4%). The study also indicated that 164,600 kg liver was condemned and destroyed. This reduced the income base for small-scale livestock farmers to about ZMW 7,407,000.00, which was equivalent to 592,560 USD.

**Conclusion:**

In conclusion, our study suggests that the prevalence of bovine fascioliasis was high resulting in a large amount of liver being condemned and destroyed, leading to economic losses for affected livestock farmers in the area. Consequently, there is a need to take the necessary measures to control the disease and create awareness among medical personnel to consider it as a differential diagnosis in all functional liver deficiencies due to the zoonotic nature of the disease.

## Background

Bovine fascioliasis is a parasitic disease of cattle caused by trematodes usually *Fasciola gigantica* and rarely *Fasciola hepatica* in the tropical countries. *Lymnaea* snails are suitable intermediate hosts for *Fasciola* spp. and lives along the river banks [1, 2]. Fascioliasis affects both domestic ruminants and humans. In humans, the disease is characterised by destruction of liver tissues and the bile duct. This causes inflammatory responses leading to hepatomegaly or cirrhotic liver accompanied by diarrhoea and anaemia [3, 4]. In herbivorous animals, it may cause enlargement and other pathological changes of the liver, which results in its condemnation. The resulting economic losses are estimated to be in the thousands of dollars, a situation that perpetuates poverty and denies the livestock farmers the considerably needed income [5, 6]. Liver damage due to immature *F. gigantica* may also predispose the animals to Black disease caused by *Clostridium novyi*, thus increasing the mortality rate [6]. Additional economic losses may be associated with expenses on anthelmintic for treatment, lower production of milk and wool, reduced weight gain, metabolic diseases and impaired fertility [7, 8]. However, this study did not consider all of these parameters. This contributes to the stagnation of livestock production and retarded economic growth in Zambia and elsewhere. This scenario was also observed by Magaji et al. 2014 in Nigeria [1] and Simwanza et al. 2012 in Zambia [8].

In the study area, animals graze on the Zambezi plain, which may be infested with *Lymnaea* snails [9]. The animals spend most of the grazing time in the plain once the water level subsides from the month of June to December. There are also agricultural activities conducted in the plain such as rice, sugar cane and vegetable growing. These activities expose human beings to infection. In the area, there were approximately 0.5 million cattle and over 95% was owned by the small-scale livestock farmers [10, 11]. Farmers keep these animals for traditional use such as paying for bride price (*lobola)* and income generation, which helps them pay for school fees for their children, buy necessary needs at home, or pay for medical bills [12].

The aim of this study was to estimate the prevalence and evaluate the direct economic loss caused by the *Fasciola* spp. in slaughtered cattle in a five-year period.

## Results

A total number of 69,152 animals was examined and 78.9% fell in the medium category [scores 3 (34.2%) and 4 (44.7%) respectively], while 16.4% was in lean category (score 2). The rest of the animals fell in the obese category (score 5) and there was no animal which fell in the emaciated category (score 1) (Table 1). All emaciated animals were rejected for slaughter at the abattoir and slaughterhouse.

**Table 1:** Category of animals examined during the study period

Year	Emaciated	Lean	Medium		Obese	Total
	1	2	3	4	5	
2013	0	2258	3596	4679	659	11,192
2014	0	1327	4526	3928	439	10,220
2015	0	3282	5229	8871	989	18,371
2016	0	2528	5743	5892	527	14,690
2017	0	1979	4567	7532	601	14,679
TOTAL	0	11,374 (16.4%)	23,661 (34.2%)	30,902 (44.7%)	3215 (4.6%)	

Of 69,152 carcasses examined, 44,511 (64.4%) were positive for *Fasciola* spp. adult worms. Among the affected animals, 24,620 (55.3%) had severely affected livers while 13,497 (30.3%) had their livers moderately affected. The rest of the carcasses (14.3%) had their livers lightly affected (Table 2).

**Table 2:** Pathological categorisation of the liver during the study

Category	2013	2014	2015	2016	2017	Total
Lightly	1389	1625	1714	1254	1412	6394 (14.4%)
Moderately	2052	1874	3682	3252	2637	13,497 (30.3%)
Severely	4107	3528	7358	4878	4749	24,620 (55.3%)
TOTAL	7548	6027	12,754	9384	8798	44,511 (64.4%)

Based on the morphological diversity, the adult liver flukes were classified according to body length, width and area. The results indicated that *F. gigantica* 38,790 (56.1%) was more prevalent than *F. hepatica* 3870 (5.6%) infecting cattle in the area. It also showed that some animals had the infection of both parasites 1851 (2.7%) (Table 3).

**Table 3:** Distribution of *Fasciola* spp. based on the body length, width and surface area of the parasite for each animal category

*Fasciola* spp.	Category of animal	Animal examined	No. of positive animals	% of positive animals
	Lean	11,374	8121	71.4
	Medium 3	23,661	12,491	52.8
*F. gigantica*	Medium 4	30,902	16,821	54.4
	Obese	3215	1357	42.2
Total		69,152	38,790	56.1
	Lean	11,374	851	7.5
	Medium 3	23,661	1327	5.6
*F. hepatica*	Medium 4	30,902	1525	4.9
	Obese	3215	167	5.2
Total		69,152	3870	5.6
	Lean	11,374	385	3.4
Mixed infection	Medium 3	23,661	593	2.5
(*F. gigantica & F. hepatica*)	Medium 4	30,902	595	1.9
	Obese	3215	278	8.6
Total		69,152	1851	2.7

Of 69,152 carcasses examined, 44,511 (64.4%) were found with the *Fasciola* spp. adult worms. In 2013, 11,192 carcasses were examined, and 67% were positive for *Fasciola* spp., in 2014, 10,220 carcasses were examined and 58% were positive. In 2015, a total of 18,371 carcasses was examined and 69.4% were positive while in 2016, a total of 14,690 slaughtered animals was inspected and 63.9% were positive and in 2017, 14,679, animals were examined and 59.9% were positive. Our study showed that a total of the 164,600 kg liver (based on an average weight of 3.698 kg/liver) was infected with the parasites, condemned and incinerated. The economic loss was estimated to be 45.00 Zambian Kwacha/kg (3.6 USD/kg) of liver multiplied by 164,600 kg and the total product of ZMW 7,407,000.00 (592,560 USD) was obtained (Table 4).

**Table 4:** The infected animals and the amount of liver condemned and incinerated from 2013 to 2017

	2013				2014				2015				2016				2017			
Months	a	b	c	d	a	b	c	d	a	b	c	d	a	b	c	d	a	b	c	d
Jan	1126	772	3088	68.6	1158	814	3256	70.3	1278	967	2901	75.7	2362	1736	5208	84.6%	938	622	2488	66.3%
Feb	987	688	2752	69.7	536	311	1244	58.0	997	679	2037	68.1	912	523	1569	64.6%	922	737	2948	79.9%
Mar	726	563	2252	77.5	714	426	1704	59.7	1131	818	2454	72.3	741	511	1533	70.8%	1027	876	3504	85.3%
Apr	903	713	2852	79.0	703	472	1888	67.1	1575	867	2601	55.0	897	528	2104	58.8%	1229	796	3184	64.8%
May	1034	729	2916	70.5	689	422	1688	61.2	1702	713	2139	41.9	920	511	2044	55.5%	1664	884	3536	53.1%
Jun	418	296	1184	70.8	623	317	1268	50.9	1299	931	2793	71.7	937	533	2132	56.9%	932	548	2192	58.8%
July	960	658	2632	68.5	658	402	1608	61.9	1422	1203	3609	84.6	1214	772	3088	63.6%	885	591	2364	66.8%
Aug	927	614	2456	66.2	774	387	1548	50.0	2083	1346	4038	64.6	887	611	2444	68.9%	1147	742	2968	64.7%
Sept	873	503	2012	57.6	889	415	1660	46.7	1328	940	2820	70.8	1048	604	2416	57.6%	1322	820	3280	62.0%
Oct	916	642	2568	70.1	1034	694	2776	67.1	2016	1781	7124	88.3	1794	1083	4332	60.4%	1510	732	2928	48.5%
Nov	973	549	2196	56.4	1168	703	2812	60.2	1276	885	3540	69.4	1384	994	3976	71.8%	1418	666	2664	47.0%
Dec	1349	821	3284	60.9	2174	664	2656	30.4	2264	1624	6496	71.7	1594	978	3912	61.4%	1685	784	3126	46.5%
Total	11,192	7548	30,192	67.4% (95%CI 67–68)	10,220	6027	24,108	59.0% (95%CI 58–60)	18,371	12,754	42,552	69.4% (95%CI 69–70)	14,690	9384	34,758	63.9% (95%CI 63–65)	14,679	8798	32,990	59.9% (95%CI 59–61)

Note

a = No. of carcasses examined

b = No. of carcasses with *Fasciola* spp.

c = Estimated Weight of Liver Condemned (kg)

d = Prevalence (%) & Confidence Interval (CI)

Direct economic loss through liver condemnation was calculated by multiplying the figures in column (c) by ZMW 45/kg

## Discussion

### Prevalence of bovine fascioliasis

We found a high prevalence of adult *Fasciola* spp. in cattle slaughtered in Mongu district, Zambia. This may indicate that these animals were infected during grazing in the Zambezi plain and surrounding areas. This could be attributed to the environmental and climatic conditions of this location that favours the survival of the intermediate hosts, the *Lymnaea* snail [1]. However, recent environmental and climatic changes due to global warming and modifications by human behaviour may probably have attributed to the increased risk of both the livestock and human populations to the disease. These might include recent urbanization, migration and development practices such as the construction of dams and roads and improving the irrigation system [9, 13–15]. As earlier reported, Zambezi plain was highly infested with the snail intermediate host, which might probably be infected with the liver flukes. Therefore, farmers were advised to deworm their animals regularly [16]. This advice most likely had fallen on deaf ears as most livestock farmers avoided visiting the veterinary clinics. This was presumably because of the charges or were busy farming or fishing or treating animals with herbal dewormers. Most farmers preferred utilising the traditional methods like herbal dewormers, which were cheaper and may fail to eliminate the parasites as suggested by Burke et al. 2009 [17]. This situation had increased the probabilities of other animals to be infected by the parasites. The infection rates from the study were lower than those described by Phiri et al. 2005 [9]. This was probably a result of little intervention conducted by the Veterinary and Public Health officers through educational awareness campaigns [10]. However, the consented effort should be consolidated and intensified to significantly reduce the prevalence rate and hence, improve the economic status of the livestock farming communities. These results are consistent with those described by Karim et al., 2015, who reported that the prevalence rate in Bangladesh was 67% in Bovine based on the abattoir investigation [16].

The results also indicated that most livers condemned were severely affected by the liver flukes. This suggests that the animals had been infected for quite a long period of time increasing the infectivity of parasites in the hosts. On the other hand, the results of our study were not consistent with those described by Bekele et al 2010 conducted in Ethiopia. The variation in pathological changes of the liver may probably be due to different factors such as exposure of the animal in the infested area with the intermediate host and period of infection, and climate-ecological conditions caused by rainfall, altitude, temperatures and suitability of the environment for the survival of the snails [12]. As described above, the river is flooded in most times of the year, which emboldens the breeding of the intermediate host and favours the transmission of the parasites to animals.

Our findings also revealed that there was a higher prevalence of fascioliasis in the lean animals in both the *Fasciola* species. This suggests that svelte animals might have been infected for a long period of time which might have affected their growth resulting in weight loss. Chronic infection with the parasites has been reported in causing poor quality carcass, reduction in growth rate and lower in productivity [5, 18]. It also indicated that obese animals were the least infected suggesting that they recently acquired the infection.

The study also revealed that *F. gigantica* was more prevalent (56.1%) than *F. hepatica* (5.6%); while a certain proportion of cattle (2.7%) harboured mixed infection (Table 3). The higher prevalence of *F. gigantica* might probably be associated with the existence of favourable ecological conditions for their intermediate hosts, the *Lymnaea* snail, as well as grazing pattern of the animals. Our observation resembles those reported by Bekele et al (2010), that *F. gigantica* was more prevalent in altitude below 1800 m above sea level as our study site is between 800 m and 1200 m above sea level [12]. These findings are consistent with those reported by Phiri et al 2007, who observed that *F. gigantica* was higher in the affected animals than *F. hepatica* [19]. In a similar study in Kenya, they reported that a more significant percentage of cattle were infected with *F. gigantica* as compared to *F. hepatica* [20]*.* Similar studies have revealed that *F. gigantica* was commonest in the Harare in Zimbabwe from the slaughtered cattle in the abattoirs [21].

### Economic impact

This study demonstrated the economic loss from fascioliasis among the livestock farmers in the country due to liver condemnation. In addition to liver condemnation, the disease causes economic losses due to the poor quality carcass, reduction in growth rate, or reduced productivity, which was not considered in this study. This results in important decreases of the household incomes of small-scale farmers, as reported elsewhere [5, 8, 18]. It is, therefore, desirable to strengthen control measures for the disease in order to minimise these losses. It is also important to educate the public about the significance of this snail-borne disease in the country. Furthermore, awareness on the disease should be created among the medical and paramedical personnel, so that they may probably consider it as a differential diagnosis in all functional liver deficiencies because the disease is zoonotic in nature.

## Conclusions

From the study, it showed that most of the carcasses were infected with the *Fasciola* species. This suggests that most of the animals acquired the parasites through grazing or drinking infested grass or water respectively. This leads to the loss of the infected livers consequently; reduce the income base of the livestock farmers.

Following these results, it was recommended that all traditional livestock farmers take their animals for a regular examination and deworming. If possible, the Department of Veterinary Services should take a compulsory deworming programme in the area.

It was also recommended that control of the intermediate hosts be intensified by using modern ways of reducing the snails. Meat inspection should be intensified in all abattoirs and slaughter slabs to monitor and improve on the health of the animals and human beings. There is equally a need to carry out the joint study with the Ministry of Health to determine the extent of the infection in humans in the study area.

## Methods

### Study site

**Fig. 1:**
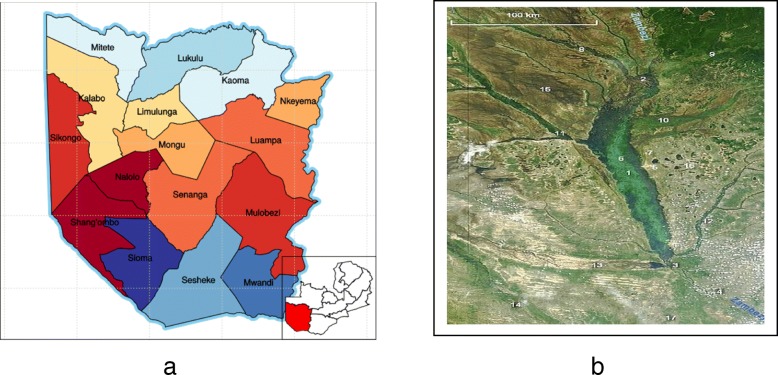
**a** Western Province Districts **b** The Zambezi Floodplain

The study took place in Mongu district in the Western province of Zambia from 2013 to 2017. The province lies between longitudes 22 degrees and 25 degrees East and latitude 13 degrees 30 min and 17 degrees 45 min South (Fig. 1a). It has an altitude of between 800 m and 1200 m above sea level. The province covers an area of about 126,386 km^2,^ which represents about 17% of the total land surface of Zambia. It consists of vast sandy, upland and the lower floodplain that covers an area of 12,950 km^2^, which is about 10% of the total land area of the province. The Zambezi River effectively bisects the province into the floodplain and the Upper land (Fig. 1b).

Western Province has sixteen districts (Fig. 1a) and Mongu is the provincial capital, which acts as the main centre for livestock trade.

The district has a dry and cold winter (April to July), hot and dry season (August to October) and hot and wet summer (November to March). It has four main ecosystems as represented by the Zambezi floodplain, the upland river valleys, wetlands and the upland forests (Fig. 1b) [22].

The annual flooding of the Zambezi floodplain controls the pattern of life for the people and livestock. The largest population is concentrated along the edges of the floodplains and they follow the transhumance subsistence type of economy. The level of the Zambezi river and its cycle of flooding controls the ecological balance and land use of the study area.

The area has a large number of animals due to agro-ecological zones that make the area suitable for livestock production and has a cattle population of approximately 500,400 [10]. The most common breed of cattle found in the province is the local one known as the Lozi breed and few cross-breeds between local and Boran cattle.

The district has two abattoirs and one slaughter slab, namely; Zambeef (Lat: − 15. 28,838, Long: 23.148439) and Starbeef (Lat: − 15.28414, Long: 23.15551) abattoirs, and Aluyi (Lat: − 15.25884, Long: 23.13359) slaughter slab, respectively.

The slaughtered animals come from different districts of the province to the abattoirs and slaughter slab in Mongu district. This is where livestock farmers find a good market with a reasonable profit due to a densely populated urban. Most of these livestock farmers were also fishermen in the Zambezi river (Fig. 1).

### Sampling techniques

#### Ante-mortem inspection and body condition scoring

In the abattoirs and slaughter slab, all animals which came for slaughter on that particular day were examined. An antemortem examination of the animals was carried out a day before or shortly prior to slaughter after obtaining verbal consent from the owners. Inspection of the animals was conducted either at rest or in motion for any obvious signs and symptoms of any disease. Any animal exhibiting physical signs of any disease was disqualified from the study. Body condition for each cattle was estimated based on Nicholson and Butterworth (1986) ranging from score 1 (emaciated) to score 5 (obese). The classes of scoring used were; emaciated (Score 1), lean (Score 2), medium (Score 3 and 4) and obese (Score 5) [12, 13, 23, 24].

#### Fluke burden and intensity of pathological lesions

Post-mortem examination of liver and associated bile duct was carefully performed by visualization and palpation of the entire organ. This was followed by transverse incision of the organ across the thin left lobe in order to macroscopically examine it [14]. Fluke burden was determined by counting the recovered adult *Fasciola* parasites, whereas pathological lesion categorisation of the affected livers was undertaken on the basis of the intensity of the lesions. Through palpation and incision of dilated or thickened bile ducts, gross pathological lesions of each liver were established and recorded. Henceforth, the affected livers were grouped into three categories as per the criteria previously described by Ogunrinade and Adegoke (1982) [14]. These were, 1) lightly affected: a quarter of the organ is affected and only one bile duct is prominently enlarged on the visceral surface of the liver, 2) moderately affected: half of the organ is affected and two or more bile ducts are hyperplastic, and 3) severely affected: almost the entire organ is involved, liver is cirrhotic and triangular in outline as the right lobe is often atrophied [25]. All the three categories resulted in the condemnation of the liver based on the guidelines on meat inspection for developing countries [26]. The condemned liver was weighed and destroyed by incineration.

#### *Fasciola* species identification

Species identification of the recovered *Fasciola* was conducted based on morphological features (including body length, body width and body area) of the parasite. These were classified either into *F. hepatica* or *F. gigantica* liver fluke species [27, 28].

#### Estimation of direct economic losses due to liver condemnation

Fascioliasis cause economic losses due to a number of reasons such as mortalities, abortions, growth retardation, reduction of milk and meat production, and condemnation of both infected livers and emaciated carcasses [14, 20]. Here, only the losses attributable to liver condemnation in slaughtered carcasses were considered. This was based on the prevalence of fascioliasis, the average weight (expressed as kg) of the liver in a mature cattle and the selling price expressed as ZMW/Kg [8, 26]. The direct economic loss (DEL) due to liver condemnation was calculated by multiplying the weight of liver condemned (WLC) by the average price of the liver/kg (APL) [20]: DEL = WLC*APL.

The average selling price of cattle liver was established through a random survey which was conducted in various meat shops during the study period. The exchange rate of 1USD to 12.5 ZMW based on information from the Bank of Zambia (BOZ), Foreign exchange department, was adopted to compute the equivalent value for USD from ZMW.

### Data analysis

Data were entered into Microsoft Excel software and the disease prevalence and corresponding confidence intervals at 95% were estimated using Epi info™ 7.0.8.0 (CDC, GA, USA). The economic or financial loss due to bovine fascioliasis was estimated using the formula described above.

## Abbreviations

BOZ: Bank of Zambia
CI: Confidence Interval
Kg: Kilogram
Lat: Latitude
Long: Longitude
ZMW: Zambian Kwacha
